# Spatial navigation deficits in early Alzheimer’s disease: the role of biomarkers and *APOE* genotype

**DOI:** 10.1007/s00415-025-13151-8

**Published:** 2025-06-02

**Authors:** Martina Laczó, Zuzana Svacova, Ondrej Lerch, Lukas Martinkovic, Monika Krejci, Zuzana Nedelska, Hana Horakova, Vaclav Matoska, Martin Vyhnalek, Jakub Hort, Michael Hornberger, Jan Laczó

**Affiliations:** 1 Memory Clinic, Department of Neurology, Second Faculty of Medicine, Charles University and Motol University Hospital, V Uvalu 84, Praha 5 – Motol, 150 06 Prague, Czechia; 2 Department of Psychology, Faculty of Arts, Charles University, Prague, Czechia; 3 Department of Clinical Biochemistry, Hematology and Immunology, Na Homolce Hospital, Prague, Czechia; 4 Norwich Medical School, University of East Anglia, Norwich, UK

**Keywords:** Allocentric navigation, Amyloid-β, Egocentric navigation, Entorhinal cortex, Hippocampus, Tau protein

## Abstract

**Background:**

Spatial navigation deficits are early symptoms of Alzheimer’s disease (AD). The apolipoprotein E (*APOE*) ε4 allele is the most important genetic risk factor for AD. This study investigated effects of *APOE* genotype on spatial navigation in biomarker-defined individuals with amnestic mild cognitive impairment (aMCI) and associations of AD biomarkers and atrophy of AD-related brain regions with spatial navigation.

**Methods:**

107 participants, cognitively normal older adults (CN, *n* = 48) and aMCI individuals stratified into AD aMCI (*n* = 28) and non-AD aMCI (*n* = 31) groups, underwent cognitive assessment, brain MRI, and spatial navigation assessment using the Virtual Supermarket Test with egocentric and allocentric tasks and a self-report questionnaire. Cerebrospinal fluid (CSF) biomarkers (amyloid-β_1–42_, phosphorylated tau_181_ and total tau) and amyloid PET imaging were assessed in aMCI participants.

**Results:**

AD aMCI participants had the highest prevalence of *APOE* ε4 carriers and worst allocentric navigation. CSF levels of AD biomarkers and atrophy in AD-related brain regions were associated with worse allocentric navigation. Between-group differences in spatial navigation and associations with AD biomarkers and regional brain atrophy were not influenced by *APOE* genotype. Self-reported navigation ability was similar across groups and unrelated to spatial navigation performance.

**Conclusions:**

These findings suggest that allocentric navigation deficits in aMCI individuals are predominantly driven by AD pathology, independent of *APOE* genotype. This highlights the role of AD pathology as measured by biomarkers, rather than genetic status, as a major factor in navigational impairment in aMCI, and emphasizes the assessment of spatial navigation as a valuable tool for early detection of AD.

**Supplementary Information:**

The online version contains supplementary material available at 10.1007/s00415-025-13151-8.

## Background

Alzheimer’s disease (AD) is the leading cause of cognitive impairment in older adults [1], and its early detection is critical for effective intervention with new disease-modifying therapies [2–4]. While AD biomarkers, such as cerebrospinal fluid (CSF) biomarkers and positron emission tomography (PET) imaging of amyloid-β (Aβ) and tau, have advanced diagnostic capabilities, their widespread use is hampered by their invasiveness, high cost and limited availability [5]. Therefore, there is an urgent need for noninvasive and cost-effective screening tools to aid in the early detection of AD. Spatial navigation assessment has emerged as a promising tool for the early detection of AD, particularly in people with mild cognitive impairment (MCI), but also in those with normal cognition [6–10]. Spatial navigation involves egocentric (body-centered) and allocentric (world-centered) strategies, both of which are affected in AD [10, 11]. Spatial navigation tests in virtual and real environments have shown the potential to distinguish people with AD pathology from those without [12–14], including amnestic MCI (aMCI) individuals with positive AD biomarkers (AD aMCI) from those with negative biomarkers (non-AD aMCI) [7, 15]. In particular, virtual tasks such as the Virtual Supermarket Test (VST) provide an ecologically valid and practical approach to assessing spatial navigation deficits in realistic scenarios [16, 17]. Previous research has linked spatial navigation performance to AD-specific biomarkers, including CSF Aβ_1–42_ and phosphorylated tau_181_ (p-tau_181_) levels [9, 15, 18], biomarkers of neurodegeneration, including CSF total tau (t-tau) and neurofilament light levels [15, 18], and atrophy in AD-related brain regions. Atrophy of the precuneus has been associated with egocentric navigation deficits, atrophy of the hippocampus and entorhinal cortex (EC), particularly the posterior hippocampus and posteromedial entorhinal cortex (pmEC), and atrophy of the basal forebrain (BF), particularly the Ch1-2 nuclei, have been associated with allocentric navigation deficits, and atrophy of the retrosplenial cortex (RSC) has been associated with deficits in both navigation strategies [15, 19]. These findings highlight the importance of spatial navigation tasks in the early diagnosis of AD and its differentiation from other amnestic neurodegenerative diseases, including the newly established clinical entity of limbic-predominant age-related TDP-43 encephalopathy (LATE) [20].

The *APOE* ε4 allele is the most important genetic risk factor for sporadic AD. It increases the risk of disease, lowers the age of onset and influences the clinical phenotype including a greater prevalence of predominant hippocampal atrophy and possibly more pronounced memory deficits [21–24]. Emerging evidence also suggests that the *APOE* ε4 allele may exacerbate impairments in certain cognitive functions, such as spatial navigation, that are sensitive to AD pathology [25, 26]. However, it remains unclear whether the *APOE* ε4 allele directly affects spatial navigation or whether its influence is primarily mediated through amyloid- and tau-related mechanisms, as it is associated with increased Aβ and tau accumulation [27–30]. Studies have shown that *APOE* ε4 carriers with aMCI had worse performance than noncarriers in both egocentric and allocentric navigation tasks [16, 31, 32]. However, these findings have not been confirmed by biomarkers, raising the question of whether these deficits reflect a true genetic influence or merely a higher prevalence of underlying AD pathology in *APOE* ε4 carriers. This ambiguity highlights the need for studies that integrate spatial navigation assessments with robust AD biomarker data to elucidate the interplay between the *APOE* ε4 allele, AD pathology, and spatial navigation deficits.

To address this knowledge gap, the present study aimed to assess: (1) the differences in spatial navigation performance in virtual egocentric and allocentric navigation tasks between participants with AD aMCI, non-AD aMCI (including those with LATE) and cognitively normal older adults, and the potential influence of *APOE* genotype on these differences; (2) the association between AD biomarkers and spatial navigation performance, and the potential influence of *APOE* genotype on this association; and (3) the association between atrophy in selected AD-related brain regions and spatial navigation deficits and the potential influence of *APOE* genotype on this association.

## Methods

### Recruitment and inclusion criteria

This study included 107 participants from the Czech Brain Aging Study (CBAS) cohort [33]. Specifically, participants with aMCI (*n* = 59) were recruited at the Memory Clinic of the Charles University, Second Faculty of Medicine, and Motol University Hospital, Prague, Czech Republic. They were referred to the Memory Clinic by general practitioners and neurologists for memory complaints reported by the participants themselves, their informants, or health professionals. Cognitively normal (CN) older adults (*n* = 48) were recruited from the University of the Third Age, senior centers, or were relatives of memory clinic participants and hospital staff. All participants underwent clinical assessment, including routine blood tests, cognitive assessment, brain magnetic resonance imaging (MRI), spatial navigation assessment, and completed a spatial navigation questionnaire. All participants with aMCI underwent biomarker assessment, including measurement of CSF Aβ_1–42_, p-tau_181_ and t-tau, or amyloid PET imaging, or both. Participants signed an informed consent form approved by the institutional ethics committee (number EK701/16). Demographic data of the participants are shown in Table 1.

i. Participants with AD aMCI (*n* = 28) met the criteria for aMCI [34] including subjectively perceived memory decline from a previously normal state, objective evidence of memory impairment (i.e., > 1.5 standard deviations [SDs] below the mean of the age-, gender- and education-adjusted norms on any memory test), maintaining independence in functional abilities (as confirmed by clinical interviews), and the absence of dementia. The participants had a positive AD biomarker signature. Specifically, 18 participants had low levels of CSF Aβ_1–42_ and 19 participants had a positive visual reading of the flutemetamol (18 F) PET scan. Of these, 9 participants had both low CSF Aβ_1–42_ levels and a positive flutemetamol (18 F) PET scan.
ii. Participants with non-AD aMCI (*n* = 31) met the criteria for aMCI [34] and had a negative AD biomarker signature. Specifically, there were 16 participants with normal levels of CSF Aβ_1–42_ and 20 participants with negative visual reading of the 18 F-flutemetamol (18 F) PET scan. Of these, 5 participants had both normal CSF Aβ_1–42_ levels and a negative flutemetamol (18 F) PET scan. 14 participants met the criteria for probable LATE [35] and 8 participants had isolated memory impairment without pronounced hippocampal atrophy and could thus have primary age-related tauopathy. The remainder of participants with non-AD aMCI did not fit into any diagnostic category.
iii. CN participants (*n* = 48) reported no cognitive complaints and had normal performance on standardized cognitive tests, adjusted for age, gender, and education. These participants had no family history of AD or other types of dementia in first-degree relatives. In addition, these participants showed no evidence of medial temporal lobe (MTL) atrophy on MRI, as visually assessed by a trained cognitive neurologist. These criteria were introduced to minimize the risk of including participants who may be at increased risk of AD, such as those with subjective cognitive decline, hippocampal atrophy or a positive family history of AD.

**Table 1:** Demographic, genetic, cognitive, neuroimaging and biomarker characteristics

	CN (*n* = 48)	non-AD aMCI (*n* = 31)	AD aMCI (*n* = 28)	Total memory clinic cohort (*n* = 107)	F/Χ^2^	*P*
Demographic characteristics						
Age (years)	68.88 (5.39)	74.42 (8.23)^a^	73.64 (5.15)^a^	71.73 (6.75)	9.08	< 0.001
Women, *n* (%)	40 (83)	13 (42)^a^	18 (64)	71 (66)	14.53	< 0.001
Education (years)	16.17 (1.95)	15.26 (2.59)	14.57 (3.06)^a^	15.49 (2.53)	3.88	0.024
MMSE (score)	29.42 (0.85)	27.39 (1.98)^a^	26.54 (2.01)^a^	28.07 (2.01)	33.49	< 0.001
Genetic characteristics						
APOE ε4 carriers (%)	12 (25)	6 (19)	17 (61)^a,b^	35 (33)	13.79	0.001
Cognitive characteristics						
GDS-15 (score)	0.94 (1.69)	3.00 (2.36)^a^	2.29 (2.36)	1.87 (2.50)	4.95	0.009
BAI (score)	5.21 (4.50)	7.97 (7.42)	6.71 (6.15)	6.40 (5.96)	1.72	0.184
AVLT 1–5 (score)	57.19 (7.06)	36.24 (6.37)^a^	32.57 (6.97)^a^	45.71 (13.24)	87.03	< 0.001
AVLT 30 (score)	11.94 (2.04)	4.69 (1.97)^a^	2.76 (3.02)^a,b^	7.83 (4.68)	118.73	< 0.001
TMT A (seconds)	38.99 (11.59)	48.43 (22.17)	57.94 (31.37)^a^	46.68 (22.59)	3.05	0.052
TMT B (seconds)	84.22 (32.59)	145.70 (63.87)^a^	154.57 (88.28)^a^	120.44 (68.55)	7.16	0.001
COWAT (score)	49.27 (10.30)	42.52 (12.73)^a^	42.89 (7.71)^a^	45.64 (10.90)	4.49	0.014
ROCFT-C (score)	31.47 (2.64)	27.77 (4.81)^a^	25.75 (5.88)^a^	28.90 (4.94)	10.56	< 0.001
ROCFT-R (score)	19.56 (5.86)	10.55 (6.54)^a^	7.05 (6.16)^a^	13.68 (8.19)	27.17	< 0.001
DSF (score)	9.42 (2.29)	8.00 (1.44)^a^	8.96 (2.05)	8.89 (2.08)	3.04	0.052
DSB (score)	6.90 (2.37)	5.48 (1.67)^a^	5.64 (1.70)	6.16 (2.12)	3.41	0.037
CDT (score)	15.40 (1.20)	14.94 (1.53)	13.46 (3.26)^a,b^	14.76 (2.15)	7.26	< 0.001
SVF Animals (score)	28.10 (5.64)	21.61 (4.75)^a^	19.54 (4.42)^a^	23.98 (6.33)	20.29	< 0.001
BNT (score)	28.23 (1.65)	26.65 (3.18)	25.11 (3.04)^a,b^	26.95 (2.84)	9.20	< 0.001
Neuroimaging characteristics^c^						
Hippocampus posterior right (volume, cm^3^)^d^	1.26 (0.14)	1.10 (0.22)^a^	1.05 (0.20)^a^	1.16 (0.20)	9.42	< 0.001
Hippocampus posterior left (volume, cm^3^) ^d^	1.32 (0.17)	1.14 (0.23)^a^	1.11 (0.17)^a^	1.21 (0.21)	8.57	< 0.001
pmEC right (volume, cm^3^) ^d^	0.36 (0.04)	0.34 (0.05)	0.31 (0.06)^a^	0.34 (0.05)	8.56	0.033
pmEC left (volume, cm^3^) ^d^	0.40 (0.04)	0.36 (0.05)^a^	0.35 (0.05)^a^	0.37 (0.05)	8.22	0.004
BF Ch1-2 (volume, cm^3^) ^d^	0.11 (0.02)	0.09 (0.03)^a^	0.10 (0.02)	0.10 (0.02)	4.96	0.009
Precuneus right (thickness, mm)	2.32 (0.13)	2.19 (0.22)^a^	2.15 (0.18)^a^	2.24 (0.18)	7.87	< 0.001
Precuneus left (thickness, mm)	2.27 (0.12)	2.18 (0.21)^a^	2.10 (0.17)^a^	2.20 (0.18)	9.11	< 0.001
Retrosplenial cortex right (thickness, mm)	2.29 (0.13)	2.17 (0.20)^a^	2.17 (0.15)	2.23 (0.17)	6.01	0.003
Retrosplenial cortex left (thickness, mm)	2.26 (0.12)	2.16 (0.22)^a^	2.17 (0.15)^a^	2.21 (0.17)	3.75	0.027
Biomarker characteristics						
CSF amyloid-β_1–42_ (pg/ml)^*e*^	N/A	1166.77 (330.16)	468.91 (84.46)^b^	796.03 (422.02)	97.13	< 0.001
CSF p-tau_181_ (pg/ml)^*e*^	N/A	67.47 (80.33)	125.00 (61.28)^b^	98.03 (75.52)	6.87	0.014
CSF total tau (pg/ml)^*e*^	N/A	366.25 (238.65)	576.81 (238.92)^b^	474.93 (258.00)	6.99	0.013
Amyloid PET positive, n (%)^*f*^	N/A	0/19 (0)	19/19 (100)^b^	19/38 (50)	38.00	< 0.001

Values are mean (SD) except for gender, APOE genotype, and amyloid PET positivity. *F*/Χ^2^ and *P* values refer to the main effect across all groups

^a^^−^^b^Significant differences between the groups based on post hoc analyses

^a^Compared to the CN group

^b^as compared to the non-AD aMCI group

^c^Based on a sample with complete brain imaging data (*n* = 102) with CN (*n* = 46), non-AD aMCI (*n* = 29) and AD aMCI (*n* = 27)

^d^Normalized to estimated total intracranial volume

^e^Based on a sample with CSF data (*n* = 34) with non-AD aMCI (*n* = 16) and AD aMCI (*n* = 18) participants

^f^Based on a sample with amyloid PET data (n = 39) with non-AD aMCI (*n* = 20) and AD aMCI (*n* = 19) participants

*CN* cognitively normal; *AD aMCI* amnestic mild cognitive impairment with positive Alzheimer’s disease biomarkers; *non-AD aMCI* amnestic mild cognitive impairment with negative Alzheimer’s disease biomarkers; *MMSE* Mini-Mental State Examination; *APOE* Apolipoprotein E; *GDS-15* Geriatric Depression Scale 15-item version; *BAI* Beck Anxiety Inventory; *LM* Logical Memory; *AVLT* Rey Auditory Verbal Learning Test; *AVLT 1–5* trials 1–5 total; *RAVLT 30* delayed word recall after 30 min; *TMT A and B* Trail Making Tests A and B; *COWAT* Controlled Oral Word Association Test (Czech version with letters N, K and P); *ROCFT-C* Rey–Osterrieth Complex Figure Test-the Copy condition; *ROCFT-R* Rey–Osterrieth Complex Figure Test-the Recall condition after 3 min; *DSF* Digit Span Forward total score; *DSB* Digit Span Backward total score; *CDT* Clock Drawing Test-Cohen’s scoring; *SVF* Semantic Verbal Fluency; *BNT* Boston Naming Test (30-item version); *pmEC* posteromedial entorhinal cortex; *BF Ch1-2* basal forebrain Ch1-2 nuclei; *CSF* cerebrospinal fluid

### Exclusion criteria

Participants with low visual acuity, gait disturbances, severe white matter hyperintensities on MRI (Fazekas score > 2 points), primary brain disorders that may affect cognitive functions, including neurological and psychiatric disorders (e.g., epilepsy, multiple sclerosis, a history of traumatic brain injury or stroke, and a history or current major psychiatric disorder), and a history of alcohol or drug abuse were not included in the study.

### Spatial navigation assessment

#### Virtual supermarket test

**Fig. 1:**
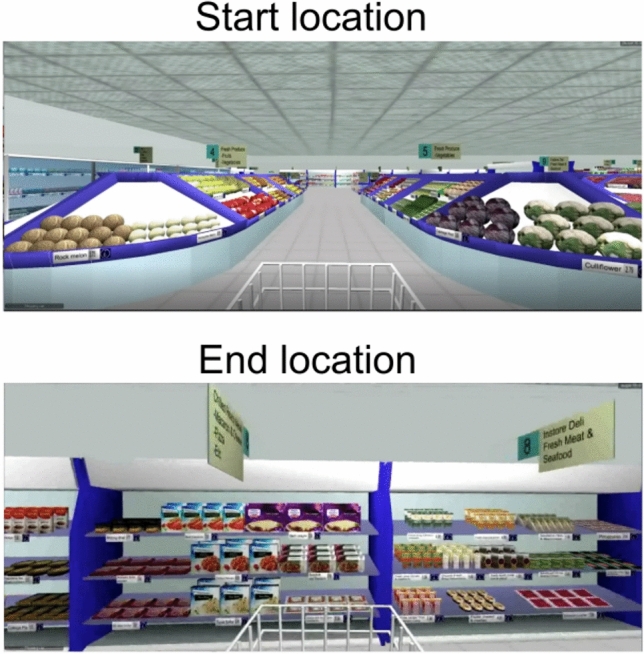
Screenshots of the Virtual Supermarket Test. The video began at the start location and followed various routes to a specified end location. Participants saw the shopping trolley in front of them as they walked through the supermarket aisles

**Fig. 2:**
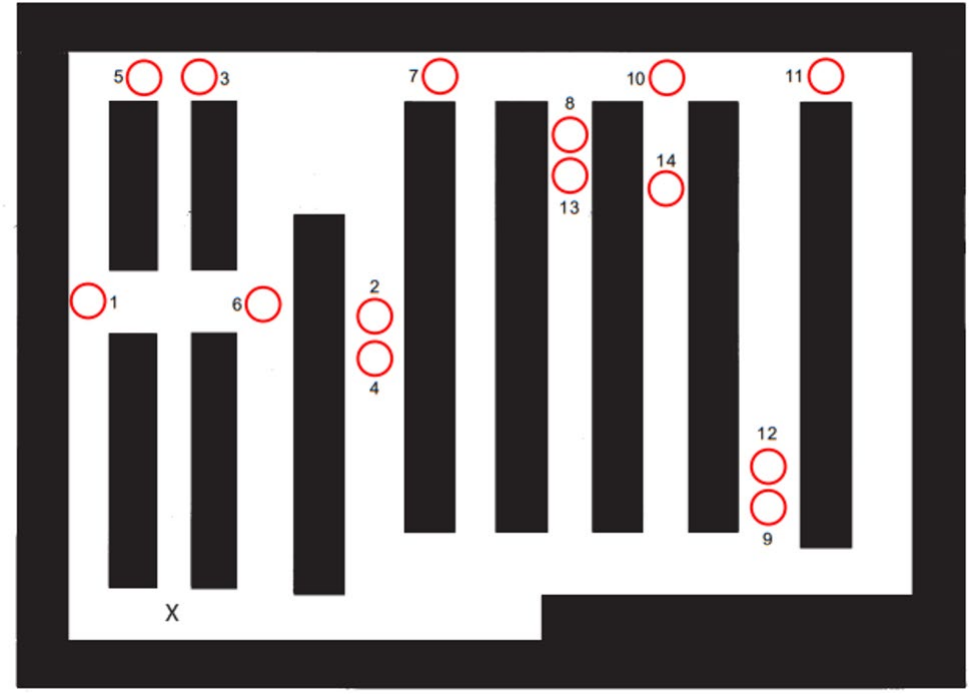
A spatial map of the supermarket, with the start location marked by an"X". This map shows the correct positions of all 14 end locations evaluated in the Allocentric Location Task. The trials were standardized in terms of both length and the number of turns (Section 1 lasted 20 s and included 3 turns, while Section 2 lasted 40 s and included 5 turns). Section 1, consisting of trials 1–7, was administered first, followed sequentially by Section 2, consisting of trials 8–14

Spatial orientation was assessed using an ecologically valid VST, which consisted of 14 video trials presented from a first-person perspective (Fig. 1) [13, 14]. Participants were instructed to imagine that they were standing behind and pushing a shopping trolley as they walked through the supermarket. In each trial, the participants travelled to a designated end location within the supermarket, making a series of 90 degree turns along the way. All trials started from the same start location, but followed different routes to reach the designated end locations. The trials were standardized in terms of both length and the number of turns (Sect. 1 lasted 20 s and included 3 turns, while Sect. 2 lasted 40 s and included 5 turns). Section 1, consisting of trials 1 to 7, was administered first, followed sequentially by Sect. 2, consisting of trials 8 to 14 (Fig. 2).

At the end of each trial, participants were asked to perform three tasks after reaching the designated end location. In the first task, the Egocentric Heading Task, the participants were instructed to indicate the direction of the start location. This was prompted by the on-screen text “In which direction is the start location?”. It is important to note that an accurate judgement of the start location could not be made by viewing it from the end location. This task required participants to perceive egocentric body rotations while navigating the supermarket. Participants were instructed to indicate only general directions, which involved distinguishing between two main components: left/right and front/back. A circular diagram representing a 360 degree field of view was provided. This diagram was divided into four quadrants (i.e., left/front, right/front, right/back, and left/back), giving the participants a total of four options to choose from.

In the second task, the Allocentric Location Task, participants were presented with a paper map of the supermarket, with the start location marked by an"X". They were asked to indicate the end location on each trial. The third task, the Allocentric Heading Task, required participants to indicate their final heading direction at the end location on the paper supermarket map. Both the second and third tasks required participants to translate their current perspective into map coordinates and orientation, thereby engaging allocentric spatial representations.

In the Egocentric and Allocentric Heading Tasks, participants received 1 point for each correct response and 0 points for incorrect responses. The mean score across 14 trials was calculated for each participant. The resulting overall mean task score ranged from 0 to 1, with higher scores indicating better task performance. For the Allocentric Location Task, performance was quantified as the distance error between the participant's indicated location and the correct location on the paper map of the supermarket. The mean distance error, measured in millimeters, was calculated over 14 trials.

No feedback was provided during the trials, and the test did not require any prior training. A short introductory video trial (10 s, 2 turns) was administered prior to testing to familiarize participants with the virtual supermarket environment and to ensure understanding of task instructions. Participants were told that they would watch a series of short video clips simulating movement to different “end” locations within the supermarket, and that on reaching each end location, they would have to make a directional judgement about the initial start location. Participants were explicitly instructed that the start location would remain fixed across all trials and that they should maintain orientation to this start location throughout each video.

### Santa Barbara Sense of Direction Scale

The Santa Barbara Sense of Direction Scale (SBSOD) was administered to participants prior to the spatial navigation assessment to measure self-reported spatial navigation ability [19]. The SBSOD was originally developed by Hegarty and colleagues [36] and consists of 15 statements that assess an individual’s spatial navigation ability in real-life situations. Sample statements included, “I very easily get lost in a new city” and “I am very good at reading maps.” Participants responded to each statement on a Likert scale ranging from 1 (strongly agree) to 7 (strongly disagree) [37]. Positively worded items, such as “I am very good at giving directions,” were reverse coded; for example, a response of 1 (strongly agree) was converted to a score of 7. Consequently, higher scores across all responses indicated greater self-reported navigation ability. The composite score was calculated as the average of all responses.

### Cognitive assessment

The following tests were used to assess cognitive function: (1) the Mini-Mental State Examination (MMSE) for global cognitive function [38]; (2) the Rey Auditory Verbal Learning Test (RAVLT)—trials 1–5 and 30-min Delayed Recall trial (RAVLT-30) for verbal memory [39]; (3) the Rey–Osterrieth Complex Figure Test (ROCFT) – the Recall condition after 3 min for nonverbal memory [40]; (4) the ROCFT – the Copy condition [40] and the Clock Drawing Test (CDT) [41] for visuospatial function; (5) the Trail Making Test (TMT) B [42] and the Phonemic Verbal Fluency—letters N, K, P for executive function [43]; (6) the Forward and Backward Digit Spans and the TMT A for attention and working memory [42]; and (7) the Boston Naming Test, a 30 odd-items version (BNT-30), and the Categorical Verbal Fluency—Animals for language [39]. The maximum time to complete TMT A and B was 180 s and 300 s, respectively, and those who were unable to complete the TMTs in a given time were scored as 181 s and 301 s, respectively. The self-report Geriatric Depression Scale, a 15-item version [44], and the Beck Anxiety Inventory [45] were administered to assess depressive and anxiety symptoms. Table 1 shows the cognitive characteristics of all study participants.

### APOE genotyping

DNA was extracted from blood (9 ml) and collected in ethylenediaminetetraacetic acid tubes using a commercial DNA extraction kit (Qiagen) according to the manufacturer's instructions. Genotyping was performed using the Idaho Technology protocol (Luna Probes Genotyping Apolipoprotein E Multiplexed Assay) for high resolution melting analysis (HRM) [32, 46]. *APOE* genotype data were available for all participants, who were further stratified into *APOE* ε4 carriers (*n* = 35) and noncarriers (*n* = 72) based on the presence of at least one *APOE* ε4 risk allele. *APOE* ε4 carriers were ε4 heterozygotes (*n* = 30) and ε4 homozygotes (*n* = 5). Table 1 shows the genetic characteristics of all study participants.

### CSF AD biomarker analysis

CSF samples were obtained by lumbar puncture in the supine position. Samples were collected in 8 mL polypropylene tubes, gently mixed, centrifuged, divided into aliquots, and stored at – 80 °C until analysis. Stored CSF samples were thawed and vortexed prior to biomarker analysis. Procedures for CSF collection, processing, and storage followed European guidelines [47]. CSF Aβ_1–42,_ p-tau_181_, and t-tau levels were analyzed using commercial enzyme-linked immunosorbent assays (ELISA) (Euroimmun) in the CSF laboratory of the Institute of Immunology and the Department of Neurology, Second Faculty of Medicine, Charles University, and Motol University Hospital. Cutoff values were set at less than 665 pg/mL for Aβ_1–42_, more than 48 pg/mL for p-tau_181_, and more than 358 pg/mL for t-tau [15]. These cutoffs were based on the internal receiver-operating characteristic (ROC) analyses and were validated against amyloid PET status in the CBAS with 79% agreement and areas under the ROC curves (AUCs) of 85 [48]. Table 1 shows the biomarker characteristics of study participants.

### Amyloid PET imaging

Dual-phase amyloid PET was used to assess Aβ positivity. PET images were acquired using a Biograph 40 TrueV HD PET/CT scanner (Siemens Healthineers AG) at the Department of Nuclear Medicine and PET Centre, Na Homolce Hospital. The participants received a single intravenous dose of flutemetamol (18 F; Vizamyl, GE Healthcare). Noncontrast, low-dose CT brain images were obtained for attenuation correction prior to the PET scans. A PET list-mode acquisition was performed in two phases: early (perfusion) and late (amyloid). The early-phase images were acquired at the time of flutemetamol (18 F) administration for 8 min and rebinned into dynamic datasets of 2 × 4 min for motion control. The late-phase images were acquired 90 min after flutemetamol (18 F) administration for a total of 10 min (2 × 5 min). The flutemetamol (18 F) PET images were visually read as positive or negative by a board-certified nuclear medicine specialist using the GM-EDGE method [49].

### Magnetic resonance imaging

MRI images were acquired using a Siemens Avanto 1.5 T scanner (Siemens AG) with a 12-channel phased-array head coil. High-resolution three-dimensional T1-weighted (3D T1w) Magnetization-Prepared Rapid Gradient Echo (MPRAGE) sequences were used with the following parameters: repetition time (TR) = 2000 ms, echo time (TE) = 3.08 ms, inversion time (TI) = 1100 ms, flip angle = 15°, 192 continuous partitions, slice thickness = 1.0 mm, and in-plane resolution = 1 mm (91). All images were visually inspected by a radiologist to exclude participants with tumours, cortical infarcts, hydrocephalus, or other major brain pathology. A trained data analyst performed quality control assessments to identify excessive motion artefacts. The 3D T1w images of sufficient quality were available for 102 participants, including CN (*n* = 46), non-AD aMCI (*n* = 29) and AD aMCI (*n* = 27) participants.

We used a previously published processing pipeline based on a CBAS template to measure hippocampal head, body and tail volumes, anterolateral EC and pmEC volumes, and estimated total intracranial volume (eTIV) [15, 50, 51]. The skull-stripped 3D T1w images were processed using statistical parametric mapping (SPM8, Wellcome Trust Center for Neuroimaging) [52] and the VBM8-toolbox (http://dbm.neuro.uni-jena.de/vbm/) implemented in MatLab R202b (MathWorks, Natick, MA). We used a CBAS template based on manual segmentation of the hippocampal and EC subregions aligned in MNI space, derived from 26 cognitively normal older adults recruited from the CBAS [33]. The CBAS template was registered and diffeomorphically warped into each participant's space using the Advanced Normalization Tools package (http://stnava.github.io/ANTs/). The resulting warp field was used to transform ROI masks of individual hippocampal and EC subregions into the participants'space. The ROI masks were then masked with a grey matter ROI and their volumes were extracted. Hippocampal body and tail volumes were summed to form posterior hippocampal volume. To reduce the number of multiple comparisons, only volumes of the posterior subregions of the hippocampus and EC (i.e., the posterior hippocampus and pmEC), which are most closely associated with spatial navigation [53, 54], were used in the statistical analyses.

The FreeSurfer image analysis suite (v7.1.0; http://surfer.nmr.mgh.harvard.edu/) was used to measure thickness of the right and left precuneus, based on the designation in the Desikan–Killiany atlas [55]. The thickness of the RSC, considered as a fused region, was derived as the area-weighted mean thickness of the ventral portions of the isthmus cingulate and posterior cingulate regions from the Desikan–Killiany atlas, based on the previous functional [56, 57] and anatomical [58] studies of the RSC.

BF volume was measured according to the published protocol [59–61]. MRI data were processed using SPM8 and the VBM8-toolbox implemented in MatLab R2023b. As in previous studies [6, 50, 51], we used a mask of the BF derived from a cytoarchitectonic map of the BF cholinergic nuclei aligned in MNI space, derived from combined histology and MRI of a postmortem brain [60, 62]. The mask included BF subregions corresponding to the Ch1-2, Ch3, Ch4p (posterior), Ch4ai (anterior and intermediate) nuclei and the nucleus subputaminalis. We nonlinearly registered images into the MNI152 template and used the resulting DARTEL parameters [62] to warp the cytoarchitectonic map into individual brain scans. Volumes of the right and left BF subregions were extracted and averaged across both hemispheres. To reduce the number of multiple comparisons, only volumes of the BF Ch1-2 nuclei, which are most closely associated with spatial navigation [6, 63], were used in the statistical analyses.

All volumes were normalized to eTIV using the previously published regression formula [64, 65]. The outputs were visually inspected for image and segmentation quality by an experienced reader blinded to clinical and biomarker data. Table 1 shows the biomarker characteristics of study participants.

### Statistical analysis

All analyses were performed in SPSS (version 28.0, IBM). The R software (R Foundation for Statistical Computing, https://www.rproject.org) was used to generate violin plots. The GLIMMPSE software (General Linear Mixed Model Power and Sample Size, http://glimmpse.samplesizeshop.org) was used to calculate power [66]. Statistical significance was set at two-tailed p < 0.05. Descriptive characteristics are presented as means and SDs for continuous variables and proportions for categorical variables. Data with non-normal distribution (i.e., AD biomarker levels) were log-transformed. Group differences in demographic and genetic characteristics were analyzed using one-way analysis of variance and chi-square tests. Group differences in cognitive performance, self-reported navigation ability, AD biomarkers, and volumes/thicknesses of selected brain regions were analyzed using general linear models (GLM). All GLM analyses were controlled for age and gender. The GLM analyses for cognitive performance and self-reported navigation ability were also controlled for years of education.

Group differences in spatial navigation performance for each VST task were analyzed using separate linear mixed models (LMM) with intercept and participant identifier as random effects, navigation trials as a repeated measure, group status, section and group status by section interaction as fixed factors, and spatial navigation score as the outcome measure, controlling for age, gender, and years of education. The supplementary LMM analyses with the non-AD aMCI group restricted to participants with LATE were also performed. Next, the MMSE score was added to the LMM analyses to account for differences in global cognition. To examine the potential effect of *APOE* genotype on group differences in spatial navigation performance and self-reported navigation ability, *APOE* genotype (1 or 2 ε4 alleles vs. no ε4 alleles) and the interaction terms with *APOE* genotype (i.e., group status by *APOE* genotype, section by *APOE* genotype and group status by section by *APOE* genotype for spatial navigation performance and group status by *APOE* genotype for self-reported navigation ability) were included in the LMM and GLM analyses, respectively. All post hoc tests were adjusted for multiple comparisons using false discovery rate (FDR) correction. To examine differences between *APOE* ε4 carriers and noncarriers in spatial navigation performance on each VST task in participants with positive and negative AD biomarkers, LMM with intercept and participant identifier as random effects, navigation trials as a repeated measure, *APOE* genotype as a fixed factor, and spatial navigation score as the outcome measure, controlling for age, gender, and years of education were used separately for the AD aMCI and non-AD aMCI groups. The power to detect significant interactions was calculated using a conditional power method, the Lawley–Hotelling trace test, a type I error rate of 0.05, data from previous VST studies [13, 14, 16], and a sample size of 107 participants. ROC analysis was used to assess the accuracy of each VST task to discriminate between the groups. AUCs with 95% CIs are reported.

The association of AD biomarkers, regional brain measures, and self-reported navigation ability with spatial navigation performance in each VST task was assessed using separate LMMs with intercept and participant identifier as random effects, navigation trials as a repeated measure, SBSOD score, level of each CSF biomarker, or volume/thickness of each selected brain region as a fixed factor, and spatial navigation score as the outcome measure, controlling for age, gender, and years of education. To examine the potential effect of *APOE* genotype on these associations, *APOE* genotype and the interaction term with the *APOE* genotype (i.e., a given fixed factor by *APOE* genotype) were included in the LMM analyses. The results are presented as unstandardized regression coefficients (*β*) with 95% CIs. FDR correction was used to adjust for multiple comparisons.

## Results

### Group characteristics

Table 1 shows the demographic, genetic, cognitive, neuroimaging and biomarker characteristics. The AD aMCI group was older, less educated and had lower MMSE scores than the CN group. The non-AD aMCI group was older, had a lower proportion of women, and had lower MMSE scores than the CN group. There were no significant differences in demographic characteristics between the AD aMCI and non-AD aMCI groups. The AD aMCI group had a higher proportion of *APOE* ε4 carriers than the non-AD aMCI and CN groups (61% vs. 19% and 25%, respectively). Both aMCI groups performed worse than the CN group on most cognitive tests, as expected. The AD aMCI and non-AD aMCI groups performed similarly on most cognitive tests, but the latter group performed better on the RAVLT-30, BNT-30, and CDT. The AD aMCI and non-AD aMCI groups had similar volumes/thicknesses of selected brain regions that were smaller than those in the CN group. The AD aMCI group had lower levels of Aβ_1–42_ and higher levels of p-tau_181_ and t-tau in the CSF than the non-AD aMCI group.

### Spatial navigation performance, self-reported navigation ability and the effect of *APOE* genotype

**Fig. 3:**
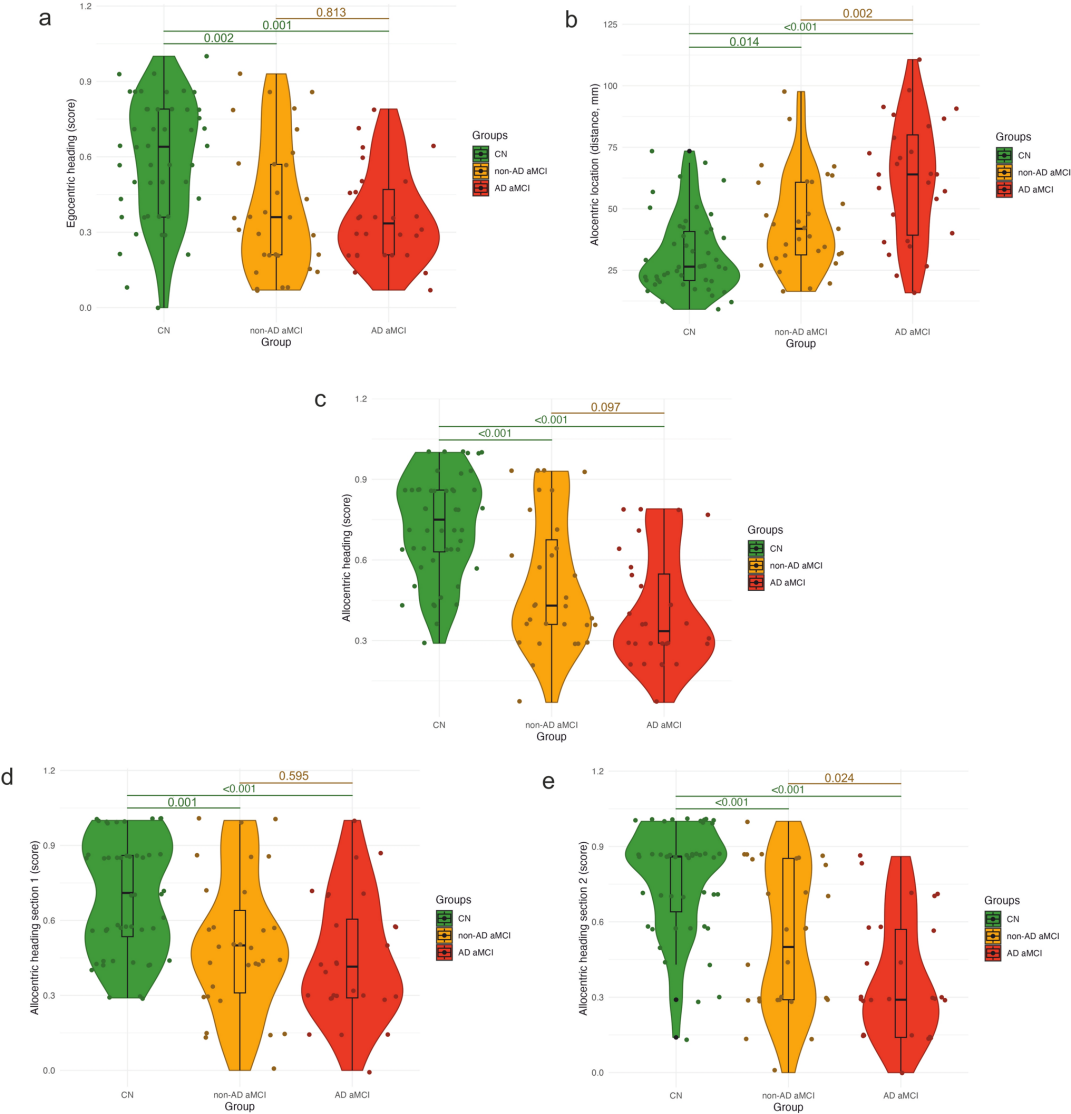
The differences in spatial navigation performance between the groups on: **a** the Egocentric Heading Task, **b** the Allocentric Location Task, **c** the Allocentric Heading Task (overall performance), **d** the Allocentric Heading Task Section 1, and **e** the Allocentric Heading Task Section 2

Figure 3 and Table 2 show the results of the differences in spatial navigation performance between the groups and Table 3 shows the results of the ROC analysis. On the Egocentric Heading Task, both aMCI groups performed worse than the CN group. There were no significant differences between the AD aMCI and non-AD aMCI groups. The effects of section and the interaction between section and group status were not significant. The task discriminated the AD aMCI and non-AD aMCI groups from the CN group with AUCs of 0.77 and 0.71, respectively. On the Allocentric Location Task, both aMCI groups performed worse than the CN group and the non-AD aMCI group was more accurate than the AD aMCI group. The effects of section and the interaction between section and group status were not significant. The task discriminated the AD aMCI and non-AD aMCI groups from the CN group with AUCs of 0.84 and 0.71, respectively, and from each other with an AUC of 0.71. On the Allocentric Heading Task, both aMCI groups performed worse than the CN group and there were no significant differences between the AD aMCI and non-AD aMCI groups. However, overall performance was more accurate in Section 1 than in Section 2 and there was a significant interaction between section and group status, showing that the non-AD aMCI group was more accurate in Section 2 than the AD aMCI group. The task discriminated the AD aMCI and non-AD aMCI groups from the CN group with AUCs of 0.87 and 0.75, respectively, and from each other in Section 2 with an AUC of 0.67. Supplementary analyses with the non-AD aMCI group restricted to participants with LATE showed that the LATE aMCI group was more accurate than the AD aMCI group on the Allocentric Location Task (see Table S1). Controlling for MMSE in the main analyses did not affect the results, except that there were no significant differences between the non-AD aMCI and CN groups on the Allocentric Location Task (see Table S2). Table 4 shows the results of the effect of *APOE* genotype on group differences in spatial navigation performance in the main analyses. We observed no significant association between *APOE* genotype, the two-way interactions of group status by *APOE* genotype and section by *APOE* genotype, or the three-way interaction of group status by section by *APOE* genotype and spatial navigation performance on any of the VST tasks. The power to detect a significant group status by *APOE* genotype interaction was ≥ 0.805. Table 5 shows the results of the effect of *APOE* genotype on spatial navigation performance for the AD aMCI and non-AD aMCI groups. No significant differences were observed between the *APOE* ε4 carriers and noncarriers on any of the VST tasks within the AD aMCI and non-AD aMCI groups.

**Table 2:** Spatial navigation performance

		*F*	*P*	Variables	Mean difference	95% Cl	*P*_post hoc_
Egocentric heading task	Diagnosis	7.660	< 0.001	CN vs. non-AD aMCI	0.184	0.070–0.299	**0.002**
				CN vs. AD aMCI	0.198	0.087–0.309	** < 0.001**
				Non-AD aMCI vs. AD aMCI	0.014	– 0.100 to 0.127	0.813
	Section	1.559	0.212				
	Diagnosis * Section	1.651	0.193				
Allocentric location task	Diagnosis	16.674	< 0.001	CN vs. non-AD aMCI	– 12.253	– 21.921 to – 2.585	**0.014**
				CN vs. AD aMCI	– 27.339	– 36.750 to – 17.928	** < 0.001**
				Non-AD aMCI vs. AD aMCI	– 15.086	– 24.694 to – 5.479	**0.002**
	Section	0.146	0.702				
	Diagnosis * Section	0.195	0.823				
Allocentric heading task	Diagnosis	19.828	< 0.001	CN vs. non-AD aMCI	0.208	0.112–0.305	** < 0.001**
				CN vs. AD aMCI	0.289	0.195–0.382	** < 0.001**
				Non-AD aMCI vs. AD aMCI	0.081	– 0.015 to 0.176	0.097
	Section	16.787	< 0.001	Section 1 vs. Section 2	0.190	0.099–0.282	** < 0.001**
	Diagnosis * Section	3.820	0.023	Section 1: CN vs. non-AD aMCI	0.180	0.070–0.290	**0.001**
				Section 1: CN vs. AD aMCI	0.210	0.102–0.319	** < 0.001**
				Section 1: non-AD aMCI vs. AD aMCI	0.030	– 0.082 to 0.143	0.595
				Section 2: CN vs. non-AD aMCI	0.236	0.126–0.347	** < 0.001**
				Section 2: CN vs. AD aMCI	0.367	0.259–0.476	** < 0.001**
				Section 2: non-AD aMCI vs. AD aMCI	0.131	0.017–0.245	**0.024**

*F* and *P* values refer to the main effect. *P*_post hoc_ values in bold were significant after false discovery rate (FDR) correction

*95% CI* 95% confidence interval; *CN* cognitively normal; *AD aMCI* amnestic mild cognitive impairment with positive Alzheimer’s disease biomarkers; *non-AD aMCI* amnestic mild cognitive impairment with negative Alzheimer’s disease biomarkers

**Table 3:** ROC analysis of spatial navigation performance

	Egocentric heading task			Allocentric location task			Allocentric heading task			Allocentric heading task – Sect. 1			Allocentric heading task – Sect. 2		
	AUC	*P*	95% Cl	AUC	*P*	95% Cl	AUC	*P*	95% Cl	AUC	*P*	95% Cl	AUC	*P*	95% Cl
CN vs. non-AD aMCI	0.709	0.001	0.588–0.831	0.714	< 0.001	0.599–0.829	0.751	< 0.001	0.632–0.870	0.697	0.001	0.575–0.818	0.748	< 0.001	0.635–0.861
CN vs. AD aMCI	0.773	< 0.001	0.668–0.879	0.842	< 0.001	0.746–0.938	0.865	< 0.001	0.780–0.950	0.766	< 0.001	0.653–0.879	0.882	< 0.001	0.805–0.959
Non-AD aMCI vs. AD aMCI	0.509	0.910	0.359–0.658	0.705	0.003	0.569–0.842	0.650	0.037	0.509–0.791	0.570	0.357	0.421–0.718	0.667	0.019	0.528–0.807

*AUC* area under the curve; *95% CI* 95% confidence interval; *CN* cognitively normal; *AD aMCI* amnestic mild cognitive impairment with positive Alzheimer’s disease biomarkers; *non-AD aMCI* amnestic mild cognitive impairment with negative Alzheimer’s disease biomarkers

**Table 4:** Spatial navigation performance and the effect of *APOE* genotype

		*F*	*P*	Variables	Mean difference	95% Cl	*P*_post hoc_
Egocentric heading task	Diagnosis	5.518	0.005	CN vs. non-AD aMCI	0.195	0.065–0.325	**0.004**
				CN vs. AD aMCI	0.161	0.044–0.279	**0.008**
				Non-AD aMCI vs. AD aMCI	– 0.033	– 0.160–0.093	0.601
	Section	1.468	0.226				
	*APOE*	3.816	0.054				
	Diagnosis * Section	1.861	0.157				
	Diagnosis * *APOE*	0.586	0.559				
	Section * *APOE*	1.551	0.214				
	Diagnosis * Section * *APOE*	1.563	0.211				
Allocentric location task	Diagnosis	12.799	< 0.001	CN vs. non-AD aMCI	– 12.466	– 23.724 to – 1.209	**0.030**
				CN vs. AD aMCI	– 26.055	– 36.287 to – 15.824	** < 0.001**
				Non-AD aMCI vs. AD aMCI	– 13.589	– 24.592 to – 2.586	**0.016**
	Section	0.372	0.542				
	APOE	0.509	0.477				
	Diagnosis * Section	0.057	0.944				
	Diagnosis * *APOE*	0.068	0.934				
	Section * *APOE*	0.053	0.818				
	Diagnosis * Section * *APOE*	0.447	0.640				
Allocentric heading task	Diagnosis	15.591	< 0.001	CN vs. non-AD aMCI	0.200	0.088–0.312	** < 0.001**
				CN vs. AD aMCI	0.281	0.179–0.382	** < 0.001**
				Non-AD aMCI vs. AD aMCI	0.080	– 0.029–0.190	0.147
	Section	15.300	< 0.001	Section 1 vs. Section 2	0.189	0.094–0.284	** < 0.001**
	*APOE*	0.034	0.854				
	Diagnosis * Section	3.272	0.039	Section 1: CN vs. non-AD aMCI	0.154	0.024–0.284	**0.021**
				Section 1: CN vs. AD aMCI	0.204	0.087–0.322	** < 0.001**
				Section 1: non-AD aMCI vs. AD aMCI	0.051	– 0.079–0.180	0.442
				Section 2: CN vs. non-AD aMCI	0.247	0.117–0.376	** < 0.001**
				Section 2: CN vs. AD aMCI	0.357	0.240–0.474	** < 0.001**
				Section 2: non-AD aMCI vs. AD aMCI	0.110	– 0.019–0.239	0.094
	Diagnosis * *APOE*	0.281	0.756				
	Section * *APOE*	1.445	0.230				
	Diagnosis * Section * *APOE*	1.673	0.189				

*F* and *P* values refer to the main effect. *P*_post hoc_ values in bold were significant after FDR correction

*95% CI* 95% confidence interval; *CN* cognitively normal; *AD aMCI* amnestic mild cognitive impairment with positive Alzheimer’s disease biomarkers; *non-AD aMCI* amnestic mild cognitive impairment with negative Alzheimer’s disease biomarkers; *APOE* apolipoprotein E

**Table 5:** Spatial navigation performance and the effect of APOE genotype in AD aMCI and non-AD aMCI groups

		AD aMCI group		Non-AD aMCI group	
		F	*P*	F	*P*
Egocentric heading task	*APOE* (ε4 carriers vs. non-carriers)	0.003	.956	2.460	.129
Allocentric location task	*APOE* (ε4 carriers vs. non-carriers)	0.060	.809	0.390	.538
Allocentric heading task	*APOE* (ε4 carriers vs. non-carriers)	1.445	.241	0.120	.732

*F* and *P* values refer to the main effect

AD aMCI, amnestic mild cognitive impairment with positive Alzheimer’s disease biomarkers; non-AD aMCI, amnestic mild cognitive impairment with negative Alzheimer’s disease biomarkers; *APOE* apolipoprotein E

There were no significant differences in self-reported navigation ability between the groups and no significant association between *APOE* genotype or the interaction of group status by *APOE* genotype and self-reported navigation ability (see Table 6). There was no significant association between self-reported navigation ability or the interaction of self-reported navigation ability by *APOE* genotype and spatial navigation performance on any of the VST tasks (see Table 7).

**Table 6:** Self-reported spatial navigation ability and the effect of *APOE* genotype

		*F*	*P*
SBSOD	Diagnosis		
		2.912	0.059
SBSOD controlled for APOE genotype	Diagnosis	1.864	0.161
	*APOE*	0.649	0.423
	Diagnosis * *APOE*	1.023	0.364

*F* and *P* values refer to the main effect

*SBSOD* Santa Barbara Sense of Direction Scale; *APOE* apolipoprotein E

**Table 7:** Association between self-reported navigation ability and spatial navigation performance, and the effect of *APOE* genotype

		Egocentric heading task			Allocentric location task			Allocentric heading task		
		*β*	*P*	95% Cl	*β*	*P*	95% Cl	*β*	*P*	95% Cl
SBSOD	SBSOD	0.008	0.739	– 0.040 to 0.056	– 2.958	0.136	– 6.861 to 0.944	0.025	0.236	– 0.017 to 0.067
SBSOD controlled for APOE genotype	SBSOD	0.368	0.546	– 0.153 to 0.0248	2.108	0.150	– 11.284 to 3.616	0.609	0.437	– 0.075 to 0.087
	*APOE*	1.935	0.168	– 0.870 to 0.153	0.393	0.532	– 56.441 to 29.357	0.062	0.804	– 0.523 to 0.407
	SBSOD * *APOE*	3.519	0.064	– 0.006 to 0.197	0.081	0.776	– 7.294 to 9.735	0.298	0.587	– 0.067 to 0.118

*β* regression coefficient; *95% CI* 95% confidence interval; *SBSOD* Santa Barbara Sense of Direction Scale; *APOE* apolipoprotein E

### Associations of AD biomarkers and regional brain measures with spatial navigation performance and the effect of *APOE* genotype

The associations of AD biomarkers and the interaction of AD biomarkers by *APOE* genotype with spatial navigation performance on each of the VST tasks are shown in Tables 8 and 9, respectively. CSF Aβ_1–42_, p-tau_181_ and t-tau levels were associated with spatial navigation performance on the Allocentric Location Task. CSF t-tau levels were associated with spatial navigation performance on the Allocentric Heading Task. No significant association was observed between AD biomarkers and spatial navigation performance on the Egocentric Heading Task. There was no significant association between the interaction of any AD biomarker by *APOE* genotype and spatial navigation performance on any of the VST tasks.

**Table 8:** Association between AD biomarkers and spatial navigation performance

	Egocentric heading task			Allocentric location task			Allocentric heading task		
	*β*	*P*	95% Cl	*β*	*P*	95% Cl	*β*	*P*	95% Cl
Amyloid-β_1–42_	0.187	0.307	– 0.183 to 0.557	– 50.849	**0.003**	– 82.717 to – 18.980	0.331	0.038	0.020 to 0.642
p-tau_181_	– 0.105	0.461	– 0.392 to 0.183	33.989	**0.013**	7.848 to 60.130	– 0.216	0.081	– 0.460 to 0.028
t-tau	– 0.213	0.231	– 0.570 to 0.144	44.782	**0.002**	17.687 to 71.877	– 0.409	**0.005**	– 0.683 to 0.134

*P* values in bold were significant after FDR correction

*β* regression coefficient; *95% CI* 95% confidence interval; *Aβ*_*1*−42_ amyloid-β_1–42_; *p-tau*_*181*_ phosphorylated tau_181_; *t-tau* total tau

**Table 9:** Associations between AD biomarkers and spatial navigation performance, and the effect of *APOE* genotype

		Egocentric heading task			Allocentric location task			Allocentric heading task		
		*β*	*p*	95% Cl	*β*	*p*	95% Cl	*β*	*p*	95% Cl
Amyloid-β_1–42_	Amyloid-β_1–42_	– 0.135	0.626	– 0.700 to 0.430	– 37.916	0.171	– 93.353 to 17.522	0.275	0.267	– 0.223 to 0.772
	APOE	– 1.079	0.334	– 3.333 to 1.176	84.970	0.437	– 136.027 to 305.966	– 0.533	0.586	– 2.520 to 1.453
	Amyloid-β_1–42_ * APOE	0.423	0.282	– 0.370 to 1.216	– 29.599	0.440	– 107.243 to 48.045	0.175	0.611	– 0.524 to 0.873
p-tau_181_	p-tau_181_	0.144	0.527	– 0.319 to 0.608	15.846	0.457	– 27.319 to 59.011	– 0.133	0.533	– 0.564 to 0.299
	APOE	0.663	0.224	– 0.432 to 1.759	– 53.145	0.295	– 155.271 to 48.980	– 0.003	0.995	– 0.024 to 1.018
	p-tau_181_ * APOE	– 0.277	0.322	– 0.842 to 0.288	24.323	0.352	– 28.457 to 77.102	0.013	0.959	– 0.514 to 0.540
t-tau	t-tau	– 0.371	0.150	– 0.886 to 0.144	31.885	0.115	– 8.403 to 72.173	– 0.558	**0.013**	– 0.985 to – 0.130
	APOE	– 0.765	0.403	– 2.624 to 1.093	– 71.652	0.319	– 216.916 to 73.611	– 1.199	0.123	– 2.746 to 0.348
	t-tau * APOE	0.345	0.325	– 0.364 to 1.054	24.632	0.368	– 30.823 to 80.087	0.468	0.114	– 0.122 to 1.058

*P* values in bold were significant after FDR correction

*β* regression coefficient; *95% CI* 95% confidence interval; *Aβ*_*1–42*_ amyloid-β_1–42_; *p-tau*_*181*_ phosphorylated tau_181_; *t-tau* total tau; *APOE* apolipoprotein E

The associations of regional brain measures and the interaction of regional brain measures by *APOE* genotype with spatial navigation performance on each of the VST tasks are shown in Tables 10, and 11, respectively. Volumes/thicknesses of all selected brain regions were associated with spatial navigation performance on the Allocentric Location Task. Volumes/thicknesses of all selected MTL regions and precuneus thickness were associated with spatial navigation performance on the Allocentric Heading Task. No significant association was observed between volumes/thicknesses of selected brain regions and spatial navigation performance on the Egocentric Heading Task. There was no significant association between the interaction of volume/thickness of any selected brain region by *APOE* genotype and spatial navigation performance on any of the VST tasks.

**Table 10:** Association between regional brain measures and spatial navigation performance

	Egocentric heading task			Allocentric location task			Allocentric heading task		
	*Β*	*P*	95% Cl	*β*	*P*	95% Cl	*β*	*P*	95% Cl
Hippocampus posterior right	0.275	0.019	0.046 to 0.503	– 27.835	**0.009**	– 48.547 to – 7.123	0.350	**0.001**	0.144 to 0.556
Hippocampus posterior left	0.272	0.018	0.048 to 0.496	– 28.717	**0.006**	– 48.961 to – 8.473	0.381	** < 0.001**	0.182 to 0.581
pmEC right	1.065	0.025	0.140 to 1.991	– 153.296	** < 0.001**	– 234.306 to – 72.286	1.149	**0.008**	0.303 to 1.996
pmEC left	0.963	0.044	0.244 to 1.901	– 124.729	**0.004**	– 208.777 to – 40.681	1.303	**0.003**	0.456 to 2.150
BF 6 Ch 1–2	1.691	0.084	– 0.230 to 3.613	– 203.639	**0.022**	– 376.997 to – 30.282	1.795	0.047	0.027 to 3.563
Precuneus right	0.117	0.354	– 0.132 to 0.367	– 46.052	** < 0.001**	– 66.927 to – 25.178	0.271	**0.019**	0.046 to 0.497
Precuneus left	0.164	0.201	– 0.089 to 0.417	– 47.476	** < 0.001**	– 68.676 to – 26.276	0.307	**0.009**	0.080 to 0.535
Retrosplenial cortex right	0.068	0.622	– 0.204 to 0.340	– 31.855	**0.010**	– 55.765 to – 7.945	0.192	0.129	– 0.057 to 0.441
Retrosplenial cortex left	0.095	0.494	– 0.180 to 0.370	– 43.500	** < 0.001**	– 67.041 to – 19.958	0.234	0.066	– 0.016 to 0.484

*P* values in bold were significant after FDR correction

*β* regression coefficient; *95% CI* 95% confidence interval; *pmEC* posteromedial entorhinal cortex; *BF Ch1-2* basal forebrain Ch1-2 nuclei

**Table 11:** Association between regional brain measures and spatial navigation performance, and the effect of *APOE* genotype

	Egocentric heading task			Allocentric location task			Allocentric heading task		
	*β*	*P*	95% Cl	*β*	*P*	95% Cl	*β*	*P*	95% Cl
Hippocampus posterior right									
Hippocampus posterior right	0.0004	0.038	0.00002 to 0.0007	– 0.039	**0.020**	– 0.071 to – 0.006	0.0005	**0.005**	0.0002 to 0.008
*APOE*	0.434	0.122	– 0.119 to 0.987	– 41.678	0.102	– 91.773 to 8.418	0.366	0.154	– 0.139 to 0.871
Hippocampus posterior right * *APOE*	– 0.0003	0.221	– 0.0008 to 0.0002	0.029	0.186	– 0.014 to 0.073	– 0.0003	0.206	– 0.0007 to 0.0002
Hippocampus posterior left									
Hippocampus posterior left	0.0003	0.137	– 0.00009 to 0.0007	– 0.037	**0.032**	– 0.071 to – 0.003	0.0005	**0.006**	0.0001 to 0.0008
*APOE*	0.220	0.438	– 0.341 to 0.781	– 33.103	0.198	– 83.813 to 17.608	0.282	0.271	– 0.224 to 0.787
Hippocampus posterior left * *APOE*	– 0.0001	0.667	– 0.0006 to 0.0004	0.020	0.337	– 0.022 to 0.062	– 0.0002	0.355	– 0.0006 to 0.0002
pmEC right									
pmEC right	0.001	0.155	– 0.0004 to 0.002	– 0.207	** < 0.001**	– 0.326 to – 0.089	0.002	**0.011**	0.0004 to 0.003
*APOE*	0.210	0.511	– 0.423 to 0.844	– 54.361	0.053	– 109.417 to 0.695	0.464	0.117	– 0.118 to 1.047
pmEC right * *APOE*	– 0.0003	0.725	– 0.002 to 0.002	0.139	0.090	– 0.022 to 0.301	– 0.001	0.162	– 0.003 to 0.0004
pmEC left									
pmEC left	0.001	0.087	–.0002 to 0.003	– 0.188	**0.006**	– 0.046 to 0.292	0.002	**0.004**	– 0.003 to 0.003
*APOE*	0.399	0.265	– 0.308 to 1.106	– 55.146	0.006	– 0.321 to – 0.055	0.542	0.098	– 0.102 to 1.186
pmEC left * *APOE*	– 0.0008	0.415	– 0.003 to 0.001	0.123	0.152	– 0.046 to 0.292	– 0.001	0.143	– 0.003 to 0.0004
BF Ch1-2									
BF Ch1-2	– 0.0005	0.743	– 0.004 to 0.003	– 0.116	0.432	– 0.406 to 0.175	0.0007	0.626	– 0.002 to 0.004
*APOE*	– 0.173	0.397	– 0.578 to 0.232	– 1.817	0.922	– 38.800 to 35.167	– 0.059	0.758	– 0.440 to 0.322
BF Ch1-2 * *APOE*	0.003	0.168	– 0.001 to 0.007	– 0.073	0.684	– 0.429 to 0.283	0.001	0.515	–.002 to 0.005
Precuneus right									
Precuneus right	0.028	0.866	– 0.299 to 0.355	– 33.845	**00.017**	– 61.545 to 6.145	0.188	0.217	– 0.113 to 0.490
*APOE*	0.004	0.994	– 1.121 to 1.130	36.389	0.451	– 59.008 to 131.786	– 0.195	0.710	– 1.231 to 0.841
Precuneus right * *APOE*	– 0.048	0.850	– 0.454 to 0.551	– 19.318	0.370	– 61.919 to – 6.145	0.113	0.629	– 0.350 to 0.576
Precuneus left									
Precuneus left	0.110	0.556	– 0.258 to 0.477	– 44.450	**0.006**	– 75.611 to 13.289	0.321	0.062	– 0.016 to 0.658
*APOE*	0.097	0.861	– 1.002 to 1.196	– 10.217	0.828	– 103.405 to 82.971	0.235	0.644	– 0.772 to 1.243
Precuneus left * APOE	0.005	0.983	– 0.495 to 0.506	1.198	0.955	– 41.209 to 43.604	– 0.080	00.730	– 0.538 to 0.379
Retrosplenial cortex right									
Retrosplenial cortex right	0.146	0.413	– 0.207 to 0.499	– 34.038	**0.033**	– 65.311 to – 2.765	0.219	0.191	– 0.111 to 0.548
*APOE*	0.617	0.301	– 0.559 to 1.793	– 30.515	0.562	– 134.501 to 73.472	0.283	0.610	– 0.814 to 1.380
Retrosplenial cortex right * *APOE*	– 0.226	0.398	– 0.753 to 0.302	9.269	0.694	– 37.347 to 55.884	– 0.093	0.707	– 0.585 to 0.399
Retrosplenial cortex left									
Retrosplenial cortex left	0.250	0.183	– 0.120 to 0.621	– 59.231	** < 0.001**	– 90.876 to – 27.587	0.465	**0.008**	0.125 to 0.804
*APOE*	0.847	0.156	– 0.329 to 2.024	– 84.497	0.098	– 184.856 to 15.862	1.143	0.038	0.067 to 2.218
Retrosplenial cortex left * *APOE*	– 0.331	0.218	– 0.861 to 0.199	33.527	0.144	– 11.712 to 78.766	33.527	0.144	– 11.712 to 78.766

*P* values in bold were significant after FDR correction

*β* regression coefficient; *95% CI* 95% confidence interval; *APOE* apolipoprotein E; *pmEC* posteromedial entorhinal cortex; *BF Ch1-2* basal forebrain Ch1-2 nuclei

## Discussion

The aim of this study was to investigate the effect of *APOE* genotype on spatial navigation in the context of AD pathology. As expected, AD aMCI participants showed worse spatial navigation performance than the non-AD aMCI participants, particularly on allocentric navigation tasks, and both aMCI groups performed worse than the CN group. *APOE* ε4 carriers were overrepresented in the AD aMCI group, but *APOE* genotype had no effect on baseline spatial navigation deficits. Instead, allocentric navigation deficits were primarily associated with AD biomarkers and atrophy in AD-related brain regions, regardless of *APOE* genotype. These findings suggest that the previously reported effect of the *APOE* ε4 allele on spatial navigation is more likely due to its contribution to Aβ and tau pathology. Importantly, self-reported navigation ability did not differ between groups, reinforcing the need for objective spatial navigation tasks, such as the VST for early detection of cognitive changes associated with AD.

The Allocentric Location Task showed the highest discriminative power between AD and non-AD aMCI participants and was particularly effective in identifying AD-related allocentric navigation deficits. The Allocentric Heading Task showed weaker discrimination between these participants, with differences only apparent on longer routes. The observed differences remained significant after controlling for global cognitive function. The Allocentric Location Task also discriminated between participants with AD aMCI and LATE aMCI, a subgroup of non-AD aMCI participants. These findings highlight the usefulness of allocentric tasks in detecting early AD and are consistent with a previous study showing greater allocentric navigation deficits in AD aMCI than in non-AD aMCI participants in a real-world task based on planning novel routes [7], and a recent study showing allocentric navigation deficits in AD aMCI, but not in non-AD aMCI, participants in a virtual city with 5 intersections [15]. Allocentric navigation deficits have also been found in individuals with preclinical AD in virtual environment studies, where CN participants with low CSF Aβ_42_ levels correctly identified fewer landmark locations out of the 20 available than those with high CSF Aβ_42_ levels [8, 9]. Allocentric navigation tasks may further identify individuals with AD in the dementia stage, as shown in a study using the VST in which AD participants performed worse on the Allocentric Heading Task, but not on the Allocentric Location Task, than participants with frontotemporal lobar degeneration (FTLD) [14]. The results of these studies suggest that the cognitive demands of specific allocentric navigation tasks are crucial for the discriminative potential of the tasks and should be tailored to the specific stages of neurodegenerative diseases.

On the Egocentric Heading Task, non-AD aMCI and AD aMCI participants performed worse than CN participants, but no significant differences were found between the aMCI groups. Previous research has shown differences in egocentric navigation between AD aMCI and non-AD aMCI individuals in virtual [15] and real-world [7] route learning tasks. Studies using the VST found that the Egocentric Heading Task discriminated between participants with AD and FTLD dementia, but they did not use biomarkers to define the underlying pathology [13, 14]. In a recent study, worse performance on the Egocentric Heading Task was shown to be more specific for vascular cognitive impairment than for cognitive impairment due to AD [67]. The lack of differences between AD aMCI and non-AD aMCI participants in the VST Egocentric Heading Task in the current study may be due to the limitations of the task and the absence of control for regional vascular lesions (e.g., white matter hyperintensities). In this task, there are four options to indicate egocentric heading direction, which may not have been sufficient to detect differences between AD aMCI and non-AD MCI individuals. The measurement of response angles on a continuous scale may be useful in future studies to improve the discrimination accuracy of the task. Future studies should also measure and control for regional white matter hyperintensities when examining differences in egocentric navigation tasks between AD aMCI and non-AD MCI individuals.

This is the first study to examine whether *APOE* genotype influences spatial navigation differences between biomarker-defined AD aMCI and non-AD aMCI participants. Our results showed that *APOE* ε4 allele had no significant effect on allocentric or egocentric spatial navigation performance at baseline. Previous studies have reported greater spatial navigation deficits in aMCI *APOE* ε4 carriers than in noncarriers, with a dose-dependent effect observed in virtual navigation tasks [31, 32]. However, these studies often lacked biomarker data, making it unclear whether spatial navigation deficits were caused by the genetic risk factor itself or by the underlying AD pathology. In CN older adults, the *APOE* ε4 allele appears to have only a minimal effect on spatial cognition, as shown in a meta-analysis and cross-sectional studies [26, 68–70], although some individual studies suggest that this allele may be associated worse performance on certain tasks [16, 25]. Longitudinal studies have demonstrated the role of the *APOE* ε4 allele in accelerating cognitive decline in Aβ-positive individuals, particularly in memory and executive function [71–73]. Our findings suggest that the *APOE* ε4 allele does not affect baseline spatial navigation in AD aMCI participants, but highlight the need for longitudinal studies to explore its potential impact on the rate of decline of spatial navigation.

Previous studies have shown that more advanced AD pathology and neurodegeneration, as measured by CSF Aβ, p-tau and neurofilament light (NfL) levels, respectively, are associated with greater spatial navigation deficits. Specifically, lower CSF Aβ_1–42_ levels were associated with worse allocentric and egocentric navigation in CN older adults [8, 17], higher CSF p-tau_181_ levels were associated with worse allocentric and egocentric navigation in aMCI individuals [15] and CN older adults [8, 9, 17], and higher NfL levels were associated with worse real-world navigation in MCI individuals [18]. Consistent with these findings, the present study found associations between lower levels of CSF Aβ_1–42_, higher levels of CSF p-tau_181_ and higher levels of CSF t-tau, as a marker of neurodegeneration, and worse navigation performance on the Allocentric Location Task. Higher levels of CSF t-tau were also associated with worse navigation performance on the Allocentric Heading Task. Aβ and p-tau accumulate early in posterior cortical and MTL regions [74, 75], the earliest sites of neurodegeneration in AD [76], which are important for egocentric to allocentric reference frame translation and allocentric processing, respectively [77–79]. Successful completion of the Allocentric Location and Allocentric Heading Tasks requires accurate processing of allocentric information along with correct translation from egocentric to allocentric perspective [14], which may underlie the observed associations between CSF AD biomarkers and navigation performance in these tasks. The nonsignificant associations between CSF Aβ_1–42_ and p-tau_181_ levels and performance on the Allocentric Heading Task may be due to the limitations of this task, which is not measured on a continuous scale. Given the accumulation of Aβ in parietal cortical regions important for egocentric navigation [80, 81], and the association between CSF Aβ_1–42_ and egocentric navigation performance in our previous study [15], we expected that lower levels of CSF Aβ_1–42_ would be associated with worse performance on the Egocentric Heading Task. The lack of the hypothesized association may be due to the limitations of this task, in which performance was not assessed on a continuous scale, potentially limiting the ability to detect differences in performance associated with Aβ pathology.

To our knowledge, no previous study has examined the effect of the *APOE* ε4 allele on the association between CSF AD biomarkers and spatial navigation. In our current study, *APOE* genotype did not modify the association between CSF AD biomarkers and spatial navigation performance in any of the VST tasks. A previous study examining the association between Aβ and memory found a moderating effect of *APOE* genotype, such that the association between CSF Aβ_1–42_ levels and memory performance was significant in *APOE* ε4 carriers but not in noncarriers [82]. These findings were not replicated in a more recent study using amyloid PET, in which *APOE* genotype had no effect on the association between cortical Aβ accumulation and memory [83]. However, there was a moderating effect of *APOE* genotype on the association between tau accumulation in the MTL regions, as measured by tau PET, and memory performance, such that higher tau levels were more strongly associated with worse memory in *APOE* ε4 carriers. In our study, we measured CSF p-tau_181_, a marker of tau pathology that is not specific to regional tau deposition [84], and found no effect of *APOE* genotype on the association with spatial navigation performance. However, we cannot exclude that the association between region-specific tau pathology, particularly in the MTL regions, and allocentric spatial navigation may be influenced by *APOE* genotype. Future studies using tau PET are needed to investigate in detail the relationships between tau pathology, spatial navigation and *APOE* genotype.

The MTL regions play an important role in allocentric navigation, where the hippocampus, particularly its posterior subregions, is involved in the accurate formation and use of cognitive maps [85] and supports fine-grained allocentric spatial representations [53]. The adjacent EC, particularly the pmEC, is important for positional and directional representations [86] and allocentric directional computations [87]. The medial parietal cortex, including the precuneus, is important for maintaining allocentric heading information during navigation [88]. Consistent with these findings, the present study showed that greater atrophy in these regions was associated with worse performance on the Allocentric Location and Allocentric Heading Tasks. We also found an association between greater atrophy of the RSC and BF Ch1-2 nuclei, and worse performance on the Allocentric Location Task. This is not surprising, as successful performance in this task relies on the translation of egocentric to allocentric reference frames, which is supported by the RSC [77]. Next, the Ch1-2 nuclei are the major source of cholinergic projections to the hippocampus [89] and their lesions cause allocentric navigation deficits [90]. The results of the present study complement and further extend previous findings on the association between atrophy in AD-related brain regions and allocentric spatial navigation deficits in aMCI individuals [6, 15, 63, 91], showing that greater atrophy is associated with less efficient cognitive mapping and estimation of allocentric directions. We found no association between regional brain atrophy and performance on the Egocentric Heading Task. Previous studies have shown that atrophy of the precuneus and RSC is associated with worse egocentric navigation in individuals with aMCI and AD dementia, respectively [13, 92]. Notably, a recent study of nearly 2000 CN older adults, including those with preclinical AD, found that smaller EC and precuneus volumes were associated with worse egocentric navigation performance on the VST [17]. However, egocentric navigation performance on the VST is also affected by white matter lesions [67], particularly those that disrupt the association pathways of the parietal cortex [93]. Our nonsignificant results may be due to a combination of the limitations of this task, which does not assess heading direction on a continuous scale, the moderate sample size, which may have reduced the statistical power to detect the associations between atrophy and egocentric navigation performance, and the lack of assessment of regional white matter hyperintensities, which may interfere with egocentric navigation performance on the VST. Future studies measuring egocentric heading direction on a continuous scale in a larger cohort of aMCI individuals and controlling for regional white matter hyperintensities are therefore needed to address these potential associations.

Studies investigating the effect of the *APOE* ε4 allele on the association between atrophy in AD-related brain regions and spatial navigation performance are lacking. In the present study, we did not find a moderating effect of *APOE* genotype on the association between atrophy in any of the selected AD-related brain regions and spatial navigation performance on any of the VST tasks. The *APOE* genotype has previously been shown to influence the rate of brain atrophy over time and regional changes in brain function during cognitive tasks. Specifically, CN older adults with the *APOE* ε4 allele had accelerated atrophy over time in the hippocampus and AD-related cortical brain regions as compared to those without the *APOE* ε4 allele [94, 95]. Next, when compared with noncarriers, the CN *APOE* ε4 carriers had reduced memory-related hippocampal activation over time, increased magnitude and the extent of brain activation during memory activation tasks in the hippocampus, parietal, and prefrontal regions, which was associated with memory decline over 2 years, and increased frontal recruitment during a demanding working memory task [96–99]. It is therefore possible that longitudinal follow-up may reveal the potential effect of *APOE* genotype on the association between regional brain atrophy and spatial navigation decline, and that functional brain changes associated with spatial navigation performance may be more susceptible to the effect of the *APOE* ε4 allele than structural brain changes. Future studies are needed to test these hypotheses.

Our previous study showed that informant-report spatial navigation questionnaires can discriminate between AD aMCI and non-AD aMCI individuals, and that their scores are strongly associated with performance in virtual and real space navigation tasks [19]. Self-report questionnaires did not discriminate between participant groups and their scores were not associated with spatial navigation performance. The only exception was the SBSOD, which discriminated between AD aMCI participants and CN older adults, and its score was weakly associated with egocentric and allocentric navigation performance in virtual and real space tasks [19]. The present study did not replicate these findings, as self-reported spatial navigation ability, as measured by the SBSOD, was similar between the groups and was not associated with allocentric or egocentric navigation performance on the VST. In addition, our study showed that *APOE* genotype did not influence nonsignificant group differences in self-reported navigation ability or its association with spatial navigation performance. These findings are consistent with previous research showing that individuals with aMCI may tend to underreport cognitive difficulties due to reduced awareness of cognitive dysfunction or an inability to accurately assess their own cognitive abilities [100].

Our study has several limitations. First, information on the biomarker profiles of the CN participants was not available. Therefore, we cannot exclude that some of them had preclinical AD. However, strict inclusion criteria were applied to minimize the likelihood of recruiting participants with preclinical AD. Second, the participants were not fully matched on demographic characteristics. In particular, the CN group was younger, more educated and had a higher proportion of women than the aMCI groups. However, all analyses were controlled for demographic characteristics to reduce the effect of these differences. Importantly, there were no differences between participants with biomarker-defined aMCI. Third, because of the small number of *APOE* ε4 homozygotes in our cohort (i.e., five *APOE* ε4/ε4 carriers), we were not able to examine a dose-dependent effect of the *APOE* ε4 allele. In addition, a moderate sample size may have reduced the statistical power to detect a possible small effect of the *APOE* genotype. Fourth, Aβ positivity or negativity was assessed using CSF Aβ_1–42_ levels, which is less accurate than using the CSF Aβ_1–42_/Aβ_1–40_ ratio. Fifth, most participants were classified into AD aMCI and non-AD aMCI groups based on CSF Aβ_1–42_ or amyloid PET results, which is less accurate than classification based on the results of both methods. Sixth, although information on Aβ was available for all aMCI participants, information on p-tau was available for a subset of them (32 of 59). Seventh, the dichotomous assessment of amyloid PET by visual reading did not allow quantification of Aβ accumulation and examination of the association between Aβ load and spatial navigation performance. Eighth, although some of the non-AD aMCI participants met the clinical criteria for probable LATE, the lack of specific biomarkers limited the ability to detect their underlying pathology. Ninth, the study was cross-sectional, so the effect of *APOE* genotype on spatial navigation decline could not be determined. However, longitudinal follow-up is ongoing.

### Practical implications and future directions

From a clinical perspective, these findings highlight the potential utility of incorporating spatial navigation tasks such as the VST into early diagnostic workflows for AD. The ecological validity of the VST, which mimics real-world navigation challenges, offers a unique advantage over traditional cognitive tests, particularly as part of a noninvasive and cost-effective screening battery. Future versions of this test, possibly adapted for remote administration via tablets or other digital platforms, could facilitate its widespread use in different clinical settings. In addition, the integration of novel, less invasive biomarkers, such as blood-based assays, together with refined spatial navigation measures (e.g., continuous angular deviation metrics for egocentric and allocentric tasks) may further improve diagnostic accuracy and clinical feasibility. Longitudinal follow-up of this cohort is an important next step. This would allow us to determine whether the *APOE* ε4 allele has a greater influence on the progression of spatial navigation deficits and the overall rate of cognitive decline over time. In addition, these efforts may help to elucidate whether the *APOE* ε4 allele interacts with AD biomarkers to accelerate disease progression or influence changes in the cognitive profile. Finally, we propose the development of a standardized spatial navigation battery, including both allocentric and egocentric tasks, as a valuable tool for the early detection of AD. Combined with advances in digital health technologies and biomarker development, such a battery holds the promise of identifying high risk individuals in the preclinical or MCI stages, allowing for timely intervention and personalized therapeutic strategies.

## Conclusions

Our study highlights several key findings that advance our understanding of spatial navigation deficits in AD and their association with *APOE* ε4 genotype. The VST allocentric navigation tasks were shown to reliably discriminate participants with AD aMCI from those with non-AD aMCI. Importantly, these deficits were primarily driven by AD pathology, as evidenced by CSF biomarkers and atrophy of AD-related brain regions, rather than the presence of the *APOE* ε4 allele. This supports the conclusion that the *APOE* ε4 allele has a limited effect on baseline spatial navigation ability but may influence longitudinal cognitive trajectories, a hypothesis that warrants further investigation.

## Supplementary Information

Below is the link to the electronic supplementary material.

Supplementary file1 (DOCX 34 KB)

## Data Availability

The datasets used and/or analyzed during the current study are available from the corresponding author upon reasonable request.
