# Supplementary material for: Spatial navigation deficits in early Alzheimer’s disease: the role of biomarkers and APOE genotype
Source: J Neurol. 2025 Jun 2;272(6):438. doi: 10.1007/s00415-025-13151-8 (PMC12130088; doi:10.1007/s00415-025-13151-8)
Supplement: Supplementary file 1 — Supplementary file1 (DOCX 34 KB) [file 415_2025_13151_MOESM1_ESM.docx]

**Supplemental table S1** Spatial navigation performance in participants with Limbic-predominant age-related TDP-43 encephalopathy

|  |  | **F** | ***P*** | **Variables** | **Mean difference** | **95% Cl** | ***P_post-hoc_*** |
| --- | --- | --- | --- | --- | --- | --- | --- |
| **Egocentric heading task** | Diagnosis | 7.845 | <.001 | CN vs. AD aMCI | 0.203 | 0.094 – 0.313 | **<.001** |
|  |  |  |  | CN vs. LATE aMCI | 0.224 | 0.073 – 0.376 | **<.001** |
|  |  |  |  | AD aMCI vs. LATE aMCI | 0.021 | -0.121 – 0.163 | .770 |
|  | Section | 0.079 | .778 |  |  |  |  |
|  | Diagnosis * Section | 1.421 | .243 |  |  |  |  |
| **Allocentric location task** | Diagnosis | 18.172 | <.001 | CN vs. AD aMCI | -27.787 | -37.189 – -18.385 | **<.001** |
|  |  |  |  | CN vs. LATE aMCI | -9.240 | -22.120 – 3.640 | .157 |
|  |  |  |  | AD aMCI vs. LATE aMCI | 18.547 | 6.466 – 30.628 | **.003** |
|  | Section | 0.314 | .575 |  |  |  |  |
|  | Diagnosis * Section | 1.402 | .247 |  |  |  |  |
| **Allocentric heading task** | Diagnosis | 21.260 | <.001 | CN vs. AD aMCI | 0.299 | 0.204 – 0.395 | **<.001** |
|  |  |  |  | CN vs. LATE aMCI | 0.292 | 0.160 – 0.423 | **<.001** |
|  |  |  |  | AD aMCI vs. LATE aMCI | -0.008 | -0.131 – 0.115 | .898 |
|  | Section | 17.589 | <.001 | Section 1 vs. Section 2 | 0.220 | 0.117 – 0.323 | **<.001** |
|  | Diagnosis * Section | 3.738 | .025 | Section 1: CN vs. AD aMCI | 0.222 | 0.112 – 0.332 | **<.001** |
|  |  |  |  | Section 1: CN vs. LATE aMCI | 0.254 | 0.106 – 0.402 | **<.001** |
|  |  |  |  | Section 1: AD aMCI vs. LATE aMCI | 0.032 | -0.111 – 0.176 | .656 |
|  |  |  |  | Section 2: CN vs. AD aMCI | 0.377 | 0.267 – 0.488 | **<.001** |
|  |  |  |  | Section 2: CN vs. LATE aMCI | 0.329 | 0.178 – 0.480 | **<.001** |
|  |  |  |  | Section 2: AD aMCI vs. LATE aMCI | -0.048 | -0.195 – 0.098 | 0.516 |

*F* and *P* values refer to the main effect. *P_post-hoc_* values in bold were significant after false discovery rate (FDR) correction.

95% CI, 95% confidence interval; CN, cognitively normal; AD aMCI, amnestic mild cognitive impairment with positive Alzheimer’s disease biomarkers; LATE aMCI, amnestic mild cognitive impairment due to Limbic-predominant age-related TDP-43 encephalopathy.

**Supplemental table S2** Spatial navigation performance controlled for the MMSE score

|  |  | **F** | **P** | **Variables** | **Mean difference** | **95% Cl** | ***P_post-hoc_*** |
| --- | --- | --- | --- | --- | --- | --- | --- |
| **Egocentric heading task** | Diagnosis | 4.165 | .018 | CN vs. non-AD aMCI | 0.164 | 0.041 – 0.287 | **.010** |
|  |  |  |  | CN vs. AD aMCI | 0.165 | 0.033 – 0.297 | **.015** |
|  |  |  |  | non-AD aMCI vs. AD aMCI | 0.001 | -0.115 – 0.118 | .986 |
|  | Section | 1.539 | .215 |  |  |  |  |
|  | Diagnosis * Section | 1.664 | .191 |  |  |  |  |
| **Allocentric location task** | Diagnosis | 8.887 | <.001 | CN vs. non-AD aMCI | -9.743 | -20.083 – 0.597 | .064 |
|  |  |  |  | CN vs. AD aMCI | -23.336 | -34.465 – -12.206 | **<.001** |
|  |  |  |  | non-AD aMCI vs. AD aMCI | -13.593 | -23.418 – -3.767 | **.007** |
|  | Section | 0.140 | .708 |  |  |  |  |
|  | Diagnosis * Section | 0.204 | .816 |  |  |  |  |
| **Allocentric heading task** | Diagnosis | 11.208 | <.001 | CN vs. non-AD aMCI | 0.188 | 0.085 – 0.292 | **<.001** |
|  |  |  |  | CN vs. AD aMCI | 0.257 | 0.146 – 0.368 | **<.001** |
|  |  |  |  | non-AD aMCI vs. AD aMCI | 0.069 | -0.030 – 0.167 | .169 |
|  | Section | 16.837 | <.001 | Section 1 vs. Section 2 | 0.191 | 0.100 – 0.282 | **<.001** |
|  | Diagnosis * Section | 3.823 | .023 | Section 1: CN vs. non-AD aMCI | 0.160 | 0.043 – 0.276 | **.007** |
|  |  |  |  | Section 1: CN vs. AD aMCI | 0.178 | 0.055 – 0.302 | **.005** |
|  |  |  |  | Section 1: non-AD aMCI vs. AD aMCI | 0.019 | -0.096 – 0.133 | .749 |
|  |  |  |  | Section 2: CN vs. non-AD aMCI | 0.217 | 0.100 – 0.333 | **<.001** |
|  |  |  |  | Section 2: CN vs. AD aMCI | 0.335 | 0.211 – 0.459 | **<.001** |
|  |  |  |  | Section 2: non-AD aMCI vs. AD aMCI | 0.118 | 0.002 – 0.234 | **.045** |

*F* and *P* values refer to the main effect. *P_post-hoc_* values in bold were significant after FDR correction.

MMSE, Mini-Mental State Examination; 95% CI, 95% confidence interval; CN, cognitively normal; AD aMCI, amnestic mild cognitive impairment with positive Alzheimer’s disease biomarkers; non-AD aMCI, amnestic mild cognitive impairment with negative Alzheimer’s disease biomarkers.
